# Maternal outcomes among women with intellectual disabilities in comparison with the general population (IDcare)

**DOI:** 10.1016/j.xagr.2025.100569

**Published:** 2025-09-05

**Authors:** Anna Axmon, Can Liu, Alessandra Grotta, Kristina Edvardsson, Magnus Sandberg

**Affiliations:** 1 Epidemiology and Bioinformatics, Division of Occupational and Environmental Medicine, Department of Laboratory Medicine, Lund University, Lund, Sweden; 2 Department of Public Health Sciences, Stockholm University, Stockholm, Sweden & Centre for Health Equity Studies, Stockholm University/Karolinska Institutet, Stockholm, Sweden & Clinical Epidemiology Division, Department of Medicine, Solna, Karolinska Institutet, Stockholm, Sweden; 3 Department of Public Health Sciences, Stockholm University, Stockholm, Sweden & Centre for Health Equity Studies, Stockholm University/Karolinska Institutet, Stockholm, Sweden; 4 Judith Lumley Centre, School of Nursing and Midwifery, La Trobe University, Melbourne, Australia; 5 Department of Health Sciences, Lund University, Lund, Sweden

**Keywords:** delivery, obstetric, intellectual disability, pregnancy complications, reproduction, routinely collected health data

## Abstract

**Background:**

Women with intellectual disabilities face significant barriers to gynecological, reproductive, antenatal, and perinatal care, which may adversely impact maternal and fetal health. Previous research indicates increased risks for gestational diabetes, pre-eclampsia, caesarean birth, preterm birth, and other complications in pregnant women with intellectual disabilities. However, studies on the reproductive health in this group remain scant, and comprehensive research on maternal and fetal health from pregnancy to the postpartum period remain missing.

**Objective(s):**

Using high-quality data from Swedish registers, the present study aims to examine a full range of maternal and fetal outcomes among birthing women with intellectual disabilities and to compare them to those of birthing women in the general population. Such knowledge is important in understanding and preventing adverse health outcomes.

**Study Design:**

This was a register study based on all women living in Skåne, Sweden on January 1^st^, 2104, with at least 1 singleton birth in 2014–2021. By linking regional and national registers, we were able to compare maternal and fetal outcomes in a cohort of women with intellectual disabilities (n=378), including a subgroup of women with diagnosis of mild intellectual disability (n=177), to outcomes among women from the general population (n=65 925). Diagnoses (i.e., outcomes) were collected from the Skåne Healthcare Register, which comprises all healthcare contacts in the Skåne region in Sweden. Poisson regression was used to estimate relative risks (RRs) with 95% confidence intervals (CIs) to quantify the association between intellectual disability and each outcome. The fully adjusted model included maternal year of birth and age at birthing, sociodemographic indicators, and obstetric comorbidities.

**Results:**

In the fully adjusted models, women with intellectual disabilities had increased risk of pre-eclampsia (RR 1.67, 95% CI 1.15–2.42), infections of the genitourinary tract (2.30, 1.67–3.16), premature rupture of membranes (2.42, 1.24–4.69 for women with mild intellectual disability), and false labor (1.27, 1.05–1.53). In crude (i.e., unadjusted) models, increased risks were also found for maternal care for known or suspected fetal abnormality and damage, maternal care for other known or suspected fetal problems, other disorders of amniotic fluid and membranes, antepartum hemorrhage, not elsewhere classified, and failed induction of labor.

**Conclusion(s):**

Pregnant women with intellectual disabilities have increased risk of several adverse maternal outcomes, with the risk for some likely driven-at least in part-by lower sociodemographic status and worse obstetric health.

**AJOG Global Reports at a Glance**

**Why was this study conducted?**

There is a lack of comprehensive studies of maternal and fetal outcomes among pregnant women with intellectual disabilities.

**Key findings**

Many increased risks among women with intellectual disabilities were partly explained by lower sociodemographic status and obstetric comorbidities. However, even after considering these factors, we found higher risks of preeclampsia, genitourinary tract infection, false labor, and premature rupture of membranes.

**What does this add to what is known?**

Women with intellectual disabilities have unique risks during pregnancy and childbirth. Our results suggest that these may to some extent be driven by sociodemography and obstetric comorbidities. More research is needed to fully understand the underlying mechanisms and how they can be reduced.

## Introduction

Intellectual disability (ID) is a neurodevelopmental disorder with significant limitations in intellectual functioning as well as adaptive behavior. The global prevalence of ID has been estimated to 1.74%.^1^ The number of women with ID who become parents is increasing,^2^ and in Sweden, it has been estimated that 225 children are born to mothers with ID each year.^3^ Women with ID face barriers to care related to sexuality and reproduction.^4^, ^5^, ^6^, ^7^ Monitoring the gaps in maternal outcomes between women with and without ID is important to achieve equality in sexual and reproductive rights, and ensure optimal healthcare for women with ID and their infants. Many risk factors for adverse pregnancy outcomes, e.g., smoking,^8^^,^^9^ overweight/obesity,^8^^,^^10^ younger age,^7^^,^^8^^,^^11^^,^^12^ and low socioeconomic status^9^^,^^10^ are more common among women with ID than among women in the general population. Therefore, antenatal services may be particularly important for this group of women and need therefore to be designed to meet their specific needs.

Recent systematic reviews^13^^,^^14^ have reported increased risks of several adverse obstetric and pregnancy outcomes, e.g., hypertensive disorders and urinary tract infections (UTIs), among pregnant women with ID. However, for a range of outcomes, such as caesarean section and pre-eclampsia, results were found to be contradictory and inconclusive. Thus, the authors conclude that there is a sparsity of literature in the field, and a need for more high-quality research. Moreover, studies assessing a comprehensive set of outcomes related to maternal health during the entire pregnancy and post-partum period are scarce.

Using high-quality data from Swedish registers, the present study aims to examine a full range of outcomes among birthing women with ID and to compare them to those of birthing women in the general population. Such knowledge is important in understanding aspects of maternal, fetal health outcomes in which women with ID.

## Materials and methods

This is a Swedish register based, cross-sectional study assessing risk of diagnoses of birth and maternal and fetal outcomes among women with ID giving birth during an 8-year study period (2014–2021), as well as comparing these risks to those among women from the general population giving birth.

### Setting

The study is set in Skåne (Scania), the southernmost county of Sweden, with a population of approximately 1.4 million in 2020 (www.scb.se), corresponding to about 10% of the total Swedish population. Antenatal care in Skåne, as well as in the rest of the country, is provided according to the national antenatal care program issued by the Swedish National Board of Health and Welfare. All antenatal care, up to and including birth, is free of charge. When the pregnancy progresses normally and without complications, care is provided by midwives. Physicians, including gynecologists and obstetricians, are only involved in the care for women who are assessed to have higher risk for complications, and when complications occur during pregnancy and childbirth. Antenatal care is normally provided at primary care facilities, and only pregnant women at particularly high risk, e.g., due to gestational diabetes, receive specialist care. The majority of women give birth at hospitals, with less than 0.05% having planned home births.^15^

### Data sources

In Sweden, service and support for people with a diagnosis of ID or autism spectrum disorder (ASD) is regulated in the Swedish Act concerning Support and Service for Persons with Certain Functional Impairments (LSS).^16^ All such service and support is reported to the Swedish National Board of Health and Welfare, and is recorded in the LSS register. Although a diagnosis of ID or ASD is required to receive support according to the LSS act, no diagnoses are specified in the LSS register.

The Skåne Healthcare Register comprises data on all healthcare contacts with public healthcare and private care providers that are under contract with the county council. For public healthcare providers, diagnoses are recorded using the International Statistical Classification of Diseases and Related Health Problems 10th Revision (ICD-10).

Statistics Sweden maintains a range of register comprising sociodemographic data on an individual level for the entire Swedish population, e.g., the Register of Education, which comprises yearly data regarding a person’s highest achieved education so far, and the Register of the Total Population, which contains data on e.g., year of birth, family type and the person’s position in the family.

### Study population

**Figure 1:**
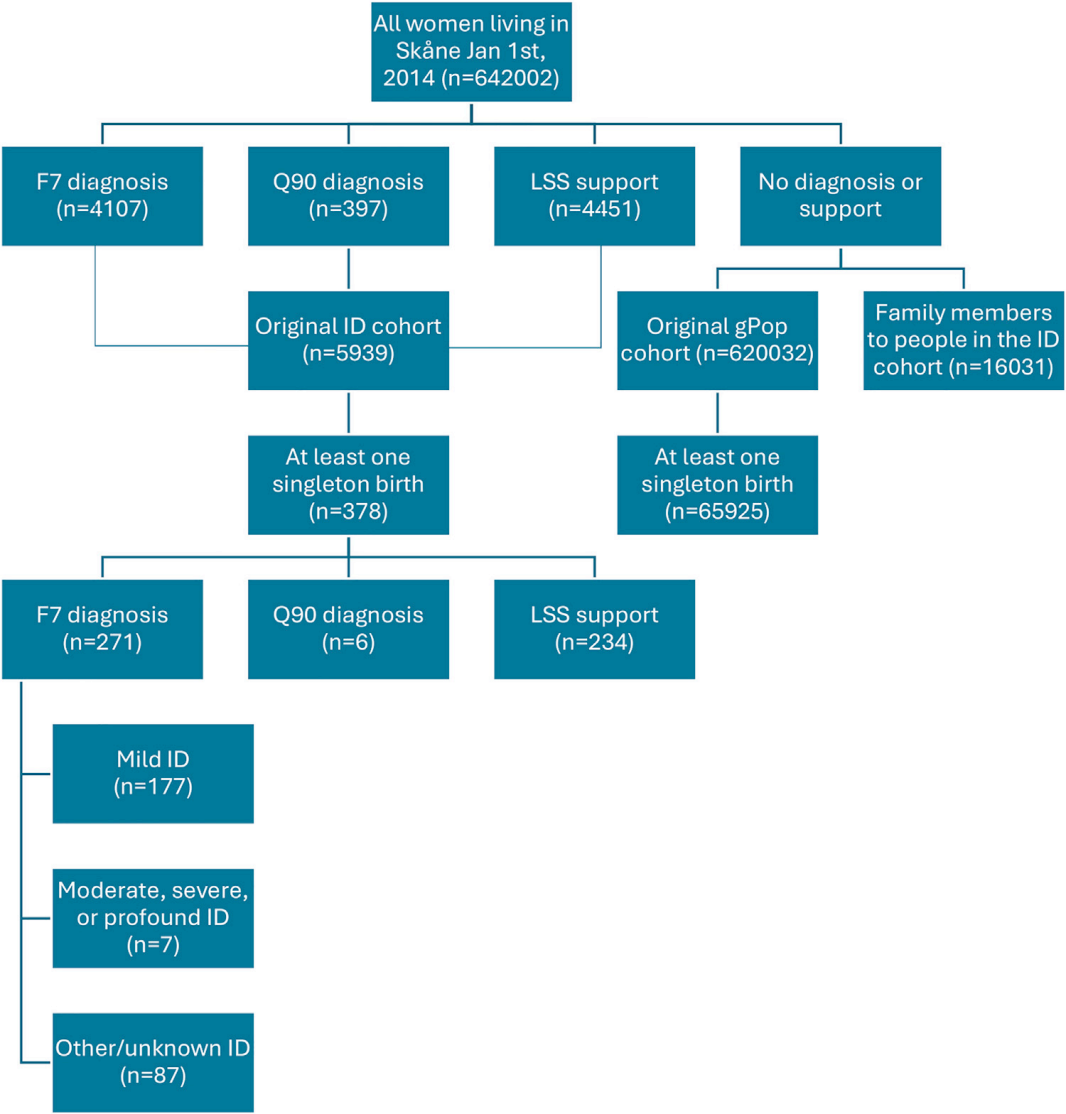
Flow chart of inclusion to and exclusion from the analyses.

This study is based on the IDcare project,^17^ in which we identified all people living in Skåne on January 1^st^, 2014. The ID cohort comprised those with at least 1 diagnosis of ID (F7 in ICD-10) or Down syndrome (Q90) during 2014–2021, or at least 1 measure of LSS support during 2007–2020, whereas the general population (gPop) cohort comprised those not in the ID cohort and not in the same household or family as a person in the ID cohort (Figure 1). Household and family members were excluded from the gPop cohort as their health and healthcare utilization have been found to differ from people without a family member with ID.^18^, ^19^, ^20^ For the present study, we identified women with at least 1 diagnosis of a singleton birth (O80–O83) in the Skåne Healthcare Register during the study period (2014–2021), resulting in 378 women from the ID cohort and 65 925 women from the gPop cohort. Moreover, we identified a subgroup within the ID cohort comprising 177 women with a diagnosis of mild ID (F70 in ICD-10) for sensitivity analyses.

Axmon. Maternal outcomes among women with intellectual disabilities. Am J Obstet Gynecol 2025.

The percentage of women with at least 1 singleton pregnancy was 6.4% in the ID cohort, 9.9% among women with mild ID, and 10.6% in the gPop cohort.

### Outcomes

Information on outcomes was collected from the Skåne Healthcare Register. These included maternal disorders [oedema, proteinuria, and hypertensive disorders in pregnancy, childbirth, and the puerperium (O10–O16), other maternal disorders predominantly related to pregnancy (O20–O29)], maternal care related to the fetus and amniotic cavity and possible delivery problems (O30–O48), complications of labor and delivery (O60–O75), and mode of delivery (O80–O83). For each diagnosis, we determined the number of women in each cohort with at least 1 occurrence.

To avoid statistical dependency, only 1 pregnancy per woman was included, the first birth during the study period. As data are left censored, this is not necessarily the woman’s first pregnancy. Non-birth diagnoses (i.e., O10–O75) were assumed to belong to the same pregnancy if they were recorded up to 40 weeks prior to the birth date or maximum 4 weeks after the birth. However, we also performed sensitivity analyses including only diagnoses up to 30 weeks prior to the birth date.

### Potential confounding factors

As potential confounding factors, we considered sociodemographic variables as well as obstetric comorbidities.

Sociodemographic variables included the woman’s own year of birth as well as her age when giving birth, cohabitation status according to the register of the total population (2014–2020), and the highest achieved education according to the register of education (2014–2020).

We determined the presence of obstetric comorbidities based on the index proposed by Leonard et al.^21^ and diagnoses from the Skåne Healthcare Register. However, we excluded diagnoses that were included as outcomes. Thus, the assessed comorbidities are pre-pregnancy chronic conditions including pulmonary hypertension, chronic renal disease, preexisting bleeding disorder, preexisting cardiac disease, HIV/AIDS, preexisting anemia, gastrointestinal disease, acute or moderate/severe asthma, substance use disorder, connective tissue or autoimmune disease, chronic hypertension, preexisting diabetes mellitus, neuromuscular disease, major mental health disorder, and thyrotoxicosis.

The women in the ID cohort (including the subgroup with mild ID) were born later (i.e., were younger during the study period; Table 1). They were also younger when giving birth, and more likely to be living alone, having only elementary school education, and having at least 1 obstetric comorbidity.

**Table 1:** Potential confounders in the gPop and ID cohorts

		gPop	ID	Mild ID
Cohabitation status [n (%)]	With partner/parents	55 879 (85)	222 (59)	95 (54)
	Single	9 958 (15)	155 (41)	81 (46)
Highest education [n (%)]	Elementary school	6 028 (9)	208 (55)	103 (59)
	Higher	59 809 (91)	169 (45)	73 (41)
Obstetric co-morbidities	None	42 677 (65)	133 (35)	59 (33)
	1	17 633 (27)	161 (43)	70 (40)
	2	4 544 (7)	60 (16)	32 (18)
	3+	1 071 (2)	24 (6)	16 (9)
Year of birth (woman) [mean (SD)]	1986 (6)	1990 (6)	1990 (6)	
Age at giving birth [mean (SD)]	31 (5)	27 (5)	27 (5)	

### Statistics

Outcomes in the ID cohort were compared to those in the gPop cohort using Poisson regression, thereby estimating relative risks (RRs) with 95% confidence intervals (CIs) for the ID cohort vs the gPop cohort.

All adjusted analyses included maternal year of birth and age at giving birth, with the extended model also including the sociodemographic indicators cohabitation status (living alone vs living with a partner or parents) and highest achieved education (elementary school or higher education [i.e., upper secondary school or post-secondary school]), and the full model additionally including number of obstetric comorbidities (none, 1, 2, or 3+). Each category of mode of delivery was treated as a distinct outcome. Thus, each mode of delivery was assessed as yes or no, with “no” comprising all other modes of delivery.

For non-birth outcomes, each ICD-10 code was assessed as a single outcome. However, in the analyses of *perineal laceration during delivery* we included only women with vaginal births, in the analyses of *failed induction of labor* we excluded women with elective caesarean section, and in the analyses of *diabetes mellitus in pregnancy* we excluded women with diagnosis of either type 1 or type 2 diabetes mellitus.

All analyses were performed in IBM SPSS Statistics 29.0. Two-tailed p-values below 0.05 were considered statistically significant. Only results based on at least 5 outcomes per group are presented.

## Results

In both cohorts and among women with mild ID, the most common mode of delivery was *single spontaneous delivery*, followed by *single delivery by caesarean section*, and *single delivery by forceps and vacuum extractor* (Table 2). Among those in the ID cohort with *single delivery by caesarean section*, 26% had *delivery by elective caesarean section* and 67% had *delivery by emergency caesarean section*, with the remaining births (7%) being either *delivery by caesarean hysterectomy, other single delivery by caesarean section*, or *delivery by caesarean section, unspecified*. The corresponding numbers among those with mild ID were 36%, 54%, and 10%, respectively, and in the gPop cohort they were 28%, 58%, and 14%, respectively. Among women with *failed induction of labor*, 79% in the ID cohort and 85% in the gPop cohort had a vaginal birth.

**Table 2:** Number (n) and percentage (%) with different pregnancy-related outcomes by ICD-10 grouping among 378 pregnant women with intellectual disability (ID), whereof 177 with mild ID, and 65 925 pregnant women from the general population (gPop)

	gPop	ID	Mild ID
Maternal disorders (O10-O16 and O20-O29)			
O13 Gestational [pregnancy-induced] hypertension	2 379 (3.6)	18 (4.8)	6 (3.4)
O14 Pre-eclampsia	2 621 (4.0)	29 (7.7)	13 (7.3)
O20 Hemorrhage in early pregnancy	3 514 (5.3)	26 (6.9)	13 (7.3)
O21 Excessive vomiting in pregnancy	4 932 (7.5)	32 (8.5)	18 (10.2)
O23 Infections of genitourinary tract in pregnancy	1 601 (2.4)	41 (10.8)	16 (9.0)
O24 Diabetes mellitus in pregnancy^a^	1 873 (2.9)	14 (3.8)	6 (3.5)
O26 Maternal care for other conditions predominantly related to pregnancy	22 293 (33.8)	144 (38.1)	68 (38.4)
Maternal care (O30-O48)			
O32 Maternal care for known or suspected malpresentation of fetus	3 642 (5.5)	17 (4.5)	10 (5.6)
O34 Maternal care for known or suspected abnormality of pelvic organs	5 277 (8.0)	20 (5.3)	9 (5.1)
O35 Maternal care for known or suspected fetal abnormality and damage	1 277 (1.9)	14 (3.7)	8 (4.5)
O36 Maternal care for other known or suspected fetal problems	11 889 (18.0)	96 (25.4)	42 (23.7)
O40 Polyhydramnios	608 (0.9)	5 (1.3)	<5
O41 Other disorders of amniotic fluid and membranes	2 495 (3.8)	23 (6.1)	6 (3.4)
O42 Premature rupture of membranes	1 335 (2.0)	12 (3.2)	9 (5.1)
O46 Antepartum hemorrhage, not elsewhere classified	2 333 (3.5)	22 (5.8)	11 (6.2)
O47 False labor	10 940 (16.6)	111 (29.4)	55 (31.1)
O48 Prolonged pregnancy	3 617 (5.5)	10 (2.6)	<5
Complications of labor and delivery (O60-O75)			
O60 Preterm labor and delivery	3 211 (4.9)	26 (6.9)	12 (6.8)
O61 Failed induction of labor^b^	13 267 (21.1)	99 (27.4)	44 (26.3)
O62 Abnormalities of forces of labor	8 472 (12.9)	44 (11.6)	20 (11.3)
O64 Obstructed labor due to malposition and malpresentation of fetus	961 (1.5)	9 (2.4)	<5
O68 Labor and delivery complicated by fetal stress [distress]	5 066 (7.7)	33 (8.7)	14 (7.9)
O70 Perineal laceration during delivery^c^	16 401 (29.9)	78 (25.0)	36 (24.2)
O71 Other obstetric trauma	1 576 (2.4)	9 (2.4)	7 (4.0)
O72 Postpartum hemorrhage	3 867 (5.9)	16 (4.2)	6 (3.4)
O75 Other complications of labor and delivery, not elsewhere classified	9 485 (14.4)	57 (15.1)	30 (16.9)
Mode of delivery (O80-O84)			
O80 Singel spontaneous delivery	49 946 (75.8)	281 (74.3)	132 (74.6)
O81 Single delivery by forceps and vacuum extractor	4 801 (7.3)	28 (7.4)	16 (9.0)
O82 Single delivery by caesarean section	11 021 (16.7)	66 (17.5)	28 (15.8)
O82.0 Delivery by elective caesarean section	3 135 (4.8)	17 (4.5)	10 (5.6)
O82.1 Delivery by emergency caesarean section	6 428 (9.8)	44 (11.6)	15 (8.5)

a Excluding women with diabetes mellitus type 1 or type 2.

b Excluding women with elective caesarean section.

c Only vaginal births.

**Figure 2:**
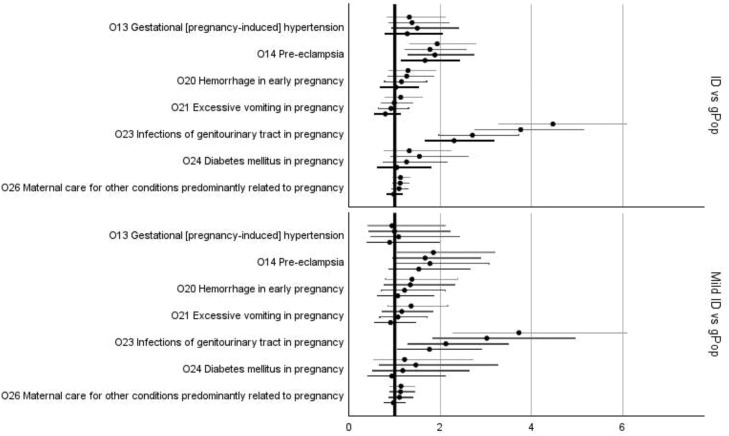
Relative risks (dots) with 95% confidence intervals (lines; top to bottom = crude, adjusted for maternal year of birth and age at birthing, additionally adjusted for education and living situations, additionally adjusted for obstetric co-morbidities) for outcomes related to maternal disorders^1^ ^1^ Oedema, proteinuria, and hypertensive disorders in pregnancy, childbirth, and the puerperium (O10-O16) and Other maternal disorders predominantly related to pregnancy (O20-O29).

**Figure 3:**
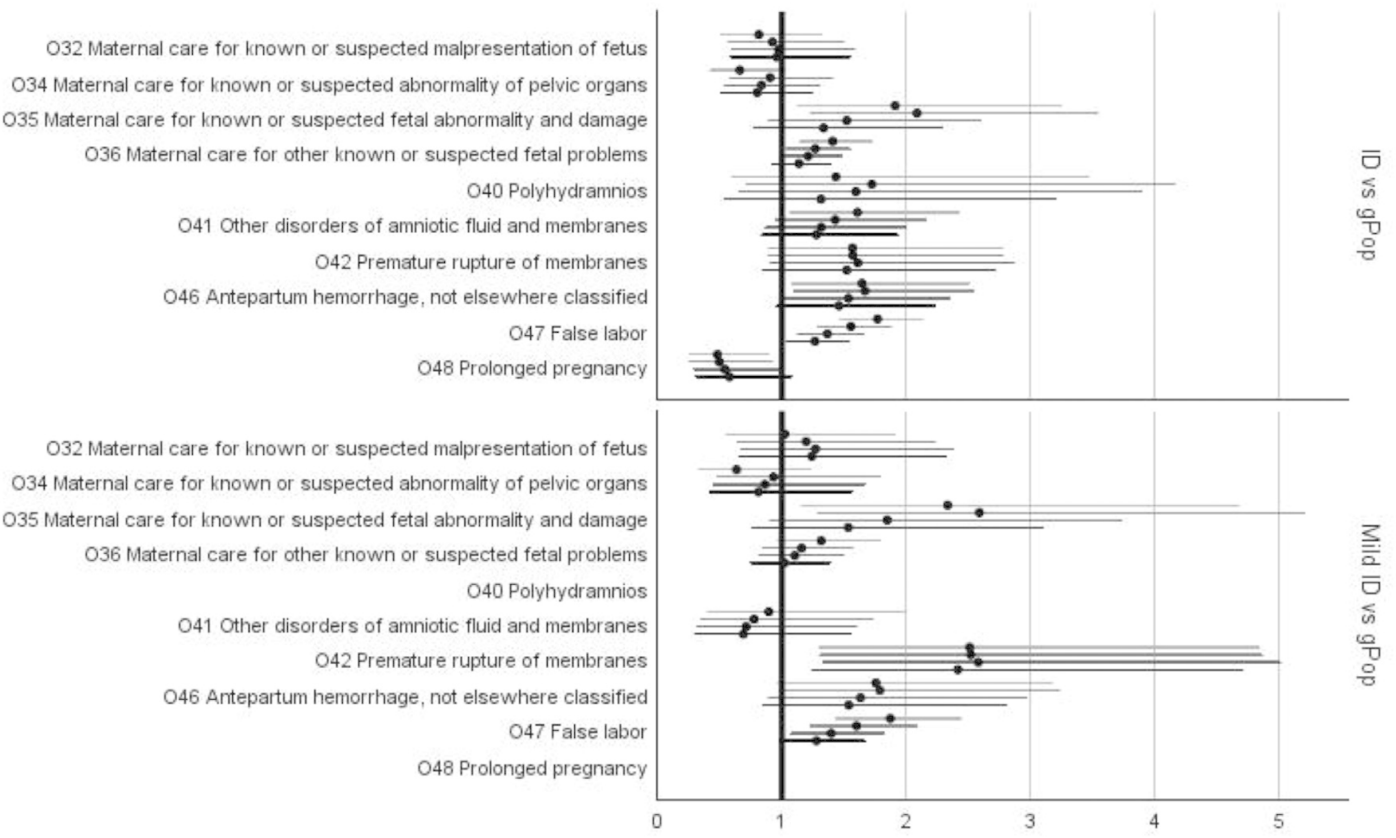
Relative risks (dots) with 95% confidence intervals (lines; top to bottom = crude, adjusted for maternal year of birth and age at birthing, additionally adjusted for education and living situations, additionally adjusted for obstetric co-morbidities) for outcomes related to maternal care^1^ ^1^ Maternal care related to the fetus and amniotic cavity and possible delivery problems (O30-O48).

**Figure 4:**
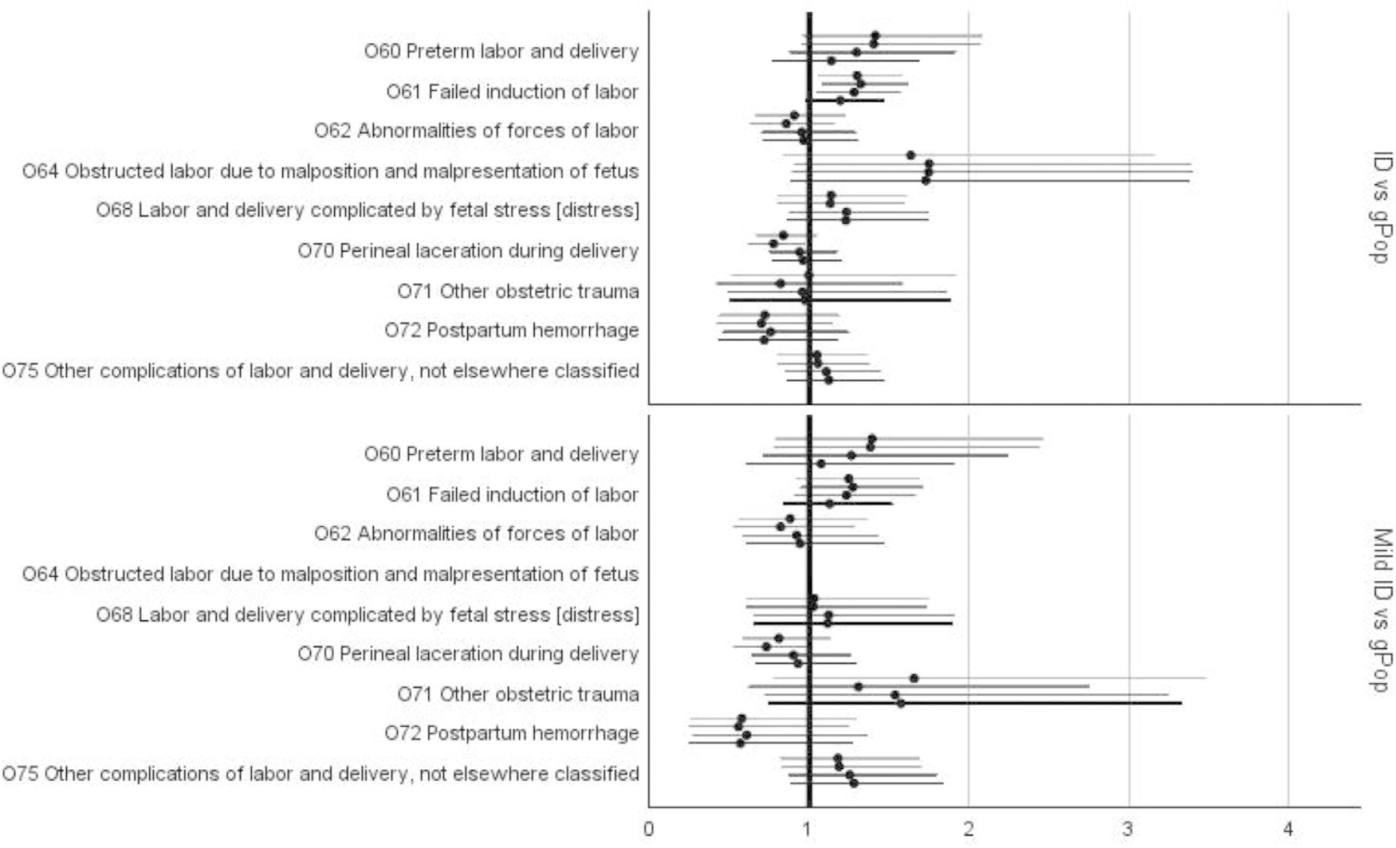
Relative risks (dots) with 95% confidence intervals (lines; top to bottom = crude, adjusted for maternal year of birth and age at birthing, additionally adjusted for education and living situations, additionally adjusted for obstetric co-morbidities) for complications of labor and delivery^1^ *^1^ Complications of labor and delivery (O60-O75)*.

**Figure 5:**
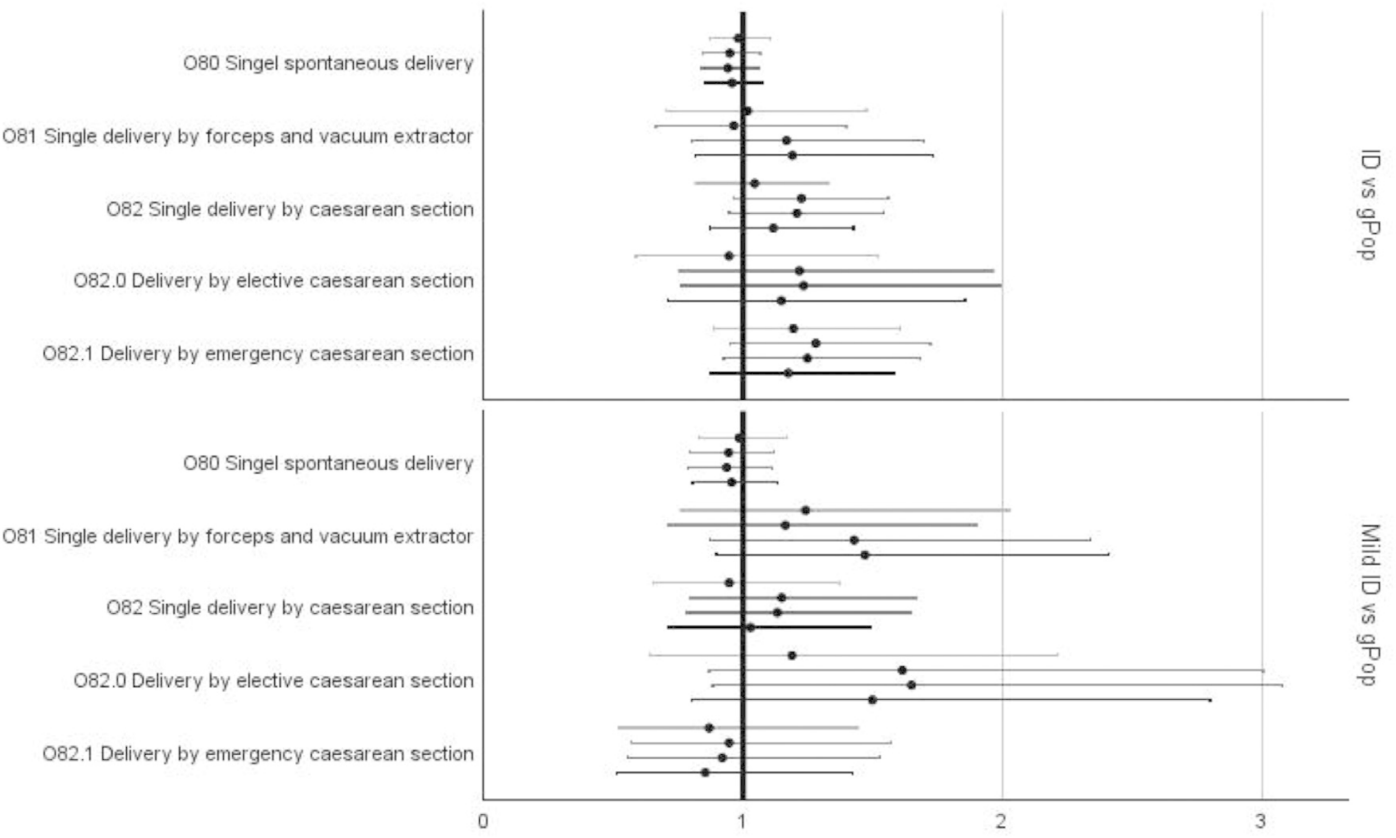
Relative risks (dots) with 95% confidence intervals (lines; top to bottom = crude, adjusted for maternal year of birth and age at birthing, additionally adjusted for education and living situations, additionally adjusted for obstetric co-morbidities) for delivery-related outcomes^1^ *^1^ Delivery (O80-O84)*.

In the fully adjusted models, statistically significant increases in risk for women in the ID cohort compared to women in the gPop cohort were found for *pre-eclampsia, infections of the genitourinary tract in pregnancy* (Figure 2; Supplement 1), and *false labor* (Figure 3; Supplement 1). The subgroup of women with mild ID had increased risk of *infections of the genitourinary tract* (Figure 2; Supplement 1) and *premature rupture of membranes* (Figure 3; Supplement 1) compared to the gPop cohort. There was no statistically significant risk difference for any of the complications of labor and delivery (Figure 4) or different modes of delivery for the women in the ID cohort compared to the women in the gPop cohort (Figure 5; Supplement 1).

Axmon. Maternal outcomes among women with intellectual disabilities. Am J Obstet Gynecol 2025.

Axmon. Maternal outcomes among women with intellectual disabilities. Am J Obstet Gynecol 2025.

Axmon. Maternal outcomes among women with intellectual disabilities. Am J Obstet Gynecol 2025.

Axmon. Maternal outcomes among women with intellectual disabilities. Am J Obstet Gynecol 2025.

In models not adjusted for sociodemographic indicators and obstetric comorbidities, there were increased risk also for women in the ID cohort for maternal care for known of suspected fetal abnormality and damage, maternal care for other known or suspected fetal problems, other disorder of amniotic fluid and membranes, and antepartum hemorrhage, not elsewhere classified. In contrast, a decreased risk was found for the ID cohort for prolonged pregnancy and failed induction of labor. However, none of these results remained statistically significant when adjusting also for sociodemographic indicators and obstetric comorbidities.

Sensitivity analyses including only diagnoses within 30 weeks prior to birth increased the risk of excessive vomiting in the fully adjusted model for women with ID (RR 0.79 vs RR 0.92) as well as for women with mild ID (RR 0.91 vs RR 1.12). No other fully adjusted risks were changed more than 10% by restricting the period for diagnoses. Moreover, none of the risk estimates changed from statistically significant to statistically non-significant, or vice versa.

## Comment

### Principal findings

Pregnant women with ID showed higher risks across the dimensions of mode of delivery, maternal disorders, maternal care, and complications during labor. For several outcomes, the higher risk among women with ID compared to the general population diminished or completely disappeared when adjusting for sociodemographic indicators and obstetric comorbidities, suggesting that these factors may be a potential driver of the increased risk.

## Results in the context of what is known

This study is the first to report on several pregnancy- and delivery-related outcomes among women with ID, including excessive vomiting in pregnancy, failed induction of labor, abnormalities of forces of labor, and perineal laceration during delivery. We are also among the first to report on antepartum and postpartum hemorrhage, infections of the genitourinary tract, premature rupture of membranes, and labor and delivery complicated by fetal stress among pregnant women with ID. Regarding the latter, our findings were generally similar to previously reported associations in other context,^13^^,^^22^^,^^23^ with minor differences possibly attributable to differences between healthcare systems. For outcomes more frequent in previous studies, our findings were consistent with previous reports regarding caesarean section,^13^^,^^22^, ^23^, ^24^ gestational hypertension,^22^^,^^25^, ^26^, ^27^, ^28^ pre-eclampsia,^7^^,^^9^^,^^25^, ^26^, ^27^, ^28^ diabetes mellitus in pregnancy,^7^^,^^9^^,^^22^^,^^26^, ^27^, ^28^, ^29^ and preterm labor and delivery^24^^,^^26^^,^^30^^,^^31^ when considering differences in context and study design.

### Clinical implications

Even after adjusting for obstetric comorbidities, we found more than double the risk for genitourinary tract infections during pregnancy among women with ID compared to women from the general population. UTIs during pregnancy are associated with a range of potentially severe consequences, such as preterm labor and birth,^32^, ^33^, ^34^ infant low birth weight,^32^^,^^33^ and maternal antenatal depressive and anxiety symptoms.^35^ Several medical risk factors for UTI are already included in the standard anamneses collected in Swedish antenatal care, including lifestyle and sociodemographic factors (e.g., smoking,^36^ level of education,^37^^,^^38^ and household income^38^ and household income^39^^,^^40^ and obesity).^41^ However, it is important that clinicians understand causes may go beyond this, and consider non-medical risk factors such as diet,^42^ sexual habits,^43^^,^^44^ and gender-based violence.^45^ For women identified as high risk, probiotics have been found to prevent UTI in general,^42^ whereas health promotion^46^ and educational^47^ interventions have been found useful to decrease the risk of UTIs during pregnancy.

Among women with mild ID, we found a more than doubled risk of premature rupture of membranes, i.e., the rupture of membranes before the onset of uterine contractions. Premature rupture before or at the limit of viability is associated with several severe obstetrical and neonatal complications.^48^ Besides UTIs,^49^^,^^50^ several other risk factors of premature rupture are more prevalent among women with ID, including a range of factors related to reproductive history (e.g., previous abortion and preterm birth), and health conditions during the pregnancy (e.g., gestational hypertension and gestational diabetes mellitus).^51^, ^52^, ^53^ Although a review from 2022 found no interventions that reduced the risk of premature ruptures,^54^ more recent research suggests that gut microbiota may play a role in the underlying mechanism.^55^^,^^56^ Therefore, dietary modifications along with vaginal probiotics may be intervention strategies to decrease the risk.^57^

Women with ID had a 67% increased risk for pre-eclampsia compared to the general population when taking obstetric comorbidities into account. Pre-eclampsia increases the risk of adverse infant health outcomes such as congenital heart disease^58^ and hypertension,^59^ as well as maternal complications later in life health, e.g., dementia^60^ and cardiovascular disease.^61^^,^^62^ Many risk factors for pre-eclampsia can be modified or controlled through lifestyle and medical interventions, e.g., overweight/obesity,^63^, ^64^, ^65^ gestational weight gain,^66^^,^^67^ diabetes,^68^^,^^69^ asthma,^70^ and UTI during pregnancy.^71^ By assessing and treating these risk factors, pre-eclampsia can be better monitored and managed in women with ID.^70^^,^^72^^,^^73^

Pregnant women with ID were more likely to have a diagnosis of false labor, i.e., contractions that are not the initiation of labor. False labor is not in itself a risk for adverse perinatal outcomes,^74^ but rather an indicator of the woman not being able to identify the onset of labor. Thus, the increased risk found for women with ID is more likely explained by a higher level of anxiety and lower levels of understanding and communication than any biological process. If clinicians are aware of this, they can make efforts to better explain different symptoms to the woman, potentially decreasing anxiety and worry for the upcoming delivery.

### Research implications

Several of the outcomes in this study have never before been studied, suggesting a need for more research. Future studies would benefit from including outcomes related to the newborn, both short term effects, e.g., birthweight, and long-term health. Also, studies concerning the pathways to different outcomes are needed to design targeted interventions to both pregnant women with ID and providers of antenatal care.

One specific suggestion for future research is for studies regarding genitourinary infections during pregnancy, including causes and prevention. Maternal genitourinary infections during pregnancy may contribute to increased risks of ID and autism in children.^22^^,^^75^^,^^76^ Although the intergenerational transmission of these conditions may be largely explained by shared familial factors, a causal effect of maternal infection on ID cannot be excluded.^77^ The extent to which this intrauterine exposure interacts with the postnatal environment remains unanswered.

### Strengths and limitations

The major strength of the present study is the use of national registers. Participation in these registers is mandatory, and thus the risk of selection bias is minimal. However, we were only able to investigate outcomes in the pregnant woman and not the newborn. Yet, the number of outcomes linked to the pregnant woman was high enough to provide a more comprehensive picture of the antenatal care of women with ID than has been done previously. A further strength of the present study is that it is conducted within a universal healthcare setting. Therefore, results are not biased from potential confounding due to financial barriers to healthcare.

Women were included in this study based on having given birth at least once during the study period. Births outside the hospital is very uncommon in Sweden, and the risk of failing to include women giving birth from either cohort is minimal. However, we have no data on when antenatal care was initiated or the amount of antenatal care received in the 2 cohorts, nor do we have information on diagnoses in antenatal care provided by private care providers. In Sweden, all people are by default listed at their geographically closest primary care provider. Therefore, switching to a private care provider requires an active choice and taking action to change your listing. It is thus reasonable to assume that women from the general population would be more likely than women with ID to seek antenatal care from a private care provider. Thus, a potential effect of not being able to include diagnoses from private care providers could be an overestimation of the risk associated with ID. On the other hand, if women with ID overall tend to seek antenatal care to a lesser degree than women in the general population, we would face an underestimation of the true risk. Future studies should investigate the amount of antenatal care used among pregnant women with ID in comparison with pregnant women from the general population, including also private care providers to assess outcomes.

## Conclusion

Pregnant women with ID have increased risk of several adverse maternal outcomes, with the risk for some likely driven-at least in part-by lower sociodemographic status and worse obstetric health. Clinicians need to be aware of the risk pattern and risk factors among these women to be able to provide antenatal care in accordance to their special needs.

## CRediT authorship contribution statement

**Anna Axmon:** Writing – review & editing, Writing – original draft, Visualization, Project administration, Methodology, Investigation, Funding acquisition, Formal analysis, Data curation, Conceptualization. **Can Liu:** Writing – review & editing, Conceptualization. **Alessandra Grotta:** Writing – review & editing, Conceptualization. **Kristina Edvardsson:** Writing – review & editing, Conceptualization. **Magnus Sandberg:** Writing – review & editing, Project administration, Funding acquisition, Conceptualization.

## Appendix Supplementary materials

Image, application 1

## Footnotes

Supplementary material associated with this article can be found in the online version at doi:10.1016/j.xagr.2025.100569.
