# Supplementary material for: Maternal outcomes among women with intellectual disabilities in comparison with the general population (IDcare)
Source: AJOG Glob Rep. 2025 Sep 5;5(4):100569. doi: 10.1016/j.xagr.2025.100569 (PMC12538695; doi:10.1016/j.xagr.2025.100569)
Supplement: Supplementary file 1 [file mmc1.docx]

*Supplement 1 Relative risks (RRs) with 95% confidence intervals (CIs) for pregnancy-related outcomes in a cohort of 382 pregnant women with intellectual disability (ID) and a subcohort of 177 pregnant women with mild ID compared to 65 925 pregnant women from the general population (gPop). Statistically significant results are marked with bold text.*

|  |  | Crude | | | Adjusted for maternal year of birth and age at birthing | | | Additionally adjusted for sociodemography^1^ | | | Additionally adjusted for obstetric co-morbidities^2^ | | |
| --- | --- | --- | --- | --- | --- | --- | --- | --- | --- | --- | --- | --- | --- |
|  |  | RR | 95% CI | | RR | 95% CI | | RR | 95% CI | | RR | 95% CI | |
| **Maternal disorders (O10-O16 and O20-O29)** |  |  |  |  |  |  |  |  |  |  |  |  |  |
| O13 Gestational [pregnancy‑induced] hypertension | ID vs gPop | 1.32 | 0.83 | 2.10 | 1.38 | 0.87 | 2.20 | 1.49 | 0.93 | 2.39 | 1.27 | 0.80 | 2.04 |
|  | Mild ID vs gPop | 0.94 | 0.42 | 2.09 | 0.99 | 0.44 | 2.21 | 1.09 | 0.49 | 2.43 | 0.89 | 0.40 | 1.99 |
| O14 Pre-eclampsia | ID vs gPop | **1.93** | **1.34** | **2.78** | **1.77** | **1.23** | **2.56** | 1.88 | **1.30** | **2.73** | **1.67** | **1.15** | **2.42** |
|  | Mild ID vs gPop | **1.85** | **1.07** | **3.19** | 1.67 | 0.97 | 2.88 | 1.77 | **1.02** | **3.07** | 1.53 | 0.88 | 2.65 |
| O20 Hemorrhage in early pregnancy | ID vs gPop | 1.29 | 0.88 | 1.90 | 1.26 | 0.86 | 1.86 | 1.15 | 0.78 | 1.70 | 1.03 | 0.69 | 1.51 |
|  | Mild ID vs gPop | 1.38 | 0.80 | 2.38 | 1.34 | 0.78 | 2.31 | 1.22 | 0.70 | 2.10 | 1.07 | 0.62 | 1.85 |
| O21 Excessive vomiting in pregnancy | ID vs gPop | 1.13 | 0.80 | 1.60 | 0.99 | 0.70 | 1.40 | 0.92 | 0.65 | 1.30 | 0.79 | 0.56 | 1.13 |
|  | Mild ID vs gPop | 1.36 | 0.86 | 2.16 | 1.15 | 0.73 | 1.83 | 1.07 | 0.67 | 1.70 | 0.91 | 0.57 | 1.45 |
| O23 Infections of genitourinary tract in pregnancy | ID vs gPop | **4.47** | **3.28** | **6.09** | **3.76** | **2.75** | **5.14** | 2.70 | **1.97** | **3.71** | **2.30** | **1.67** | **3.16** |
|  | Mild ID vs gPop | **3.72** | **2.27** | **6.09** | **3.02** | **1.84** | **4.95** | 2.12 | **1.29** | **3.49** | **1.76** | **1.07** | **2.90** |
| O24 Diabetes mellitus in pregnancy^3^ | ID vs gPop | 1.32 | 0.78 | 2.23 | 1.54 | 0.91 | 2.61 | 1.26 | 0.74 | 2.14 | 1.05 | 0.61 | 1.78 |
|  | Mild ID vs gPop | 1.22 | 0.55 | 2.71 | 1.46 | 0.66 | 3.26 | 1.18 | 0.53 | 2.63 | 0.94 | 0.42 | 2.10 |
| O26 Maternal care for other conditions predominantly related to pregnancy | ID vs gPop | 1.13 | 0.96 | 1.33 | 1.12 | 0.95 | 1.32 | 1.09 | 0.93 | 1.29 | 0.98 | 0.83 | 1.15 |
|  | Mild ID vs gPop | 1.14 | 0.90 | 1.44 | 1.13 | 0.89 | 1.43 | 1.10 | 0.87 | 1.40 | 0.97 | 0.76 | 1.23 |
| **Maternal care (O30-O48)** |  |  |  |  |  |  |  |  |  |  |  |  |  |
| O32 Maternal care for known or suspected malpresentation of fetus | ID vs gPop | 0.81 | 0.51 | 1.31 | 0.93 | 0.57 | 1.49 | 0.97 | 0.60 | 1.58 | 0.96 | 0.59 | 1.55 |
|  | Mild ID vs gPop | 1.02 | 0.55 | 1.90 | 1.20 | 0.64 | 2.22 | 1.27 | 0.68 | 2.37 | 1.24 | 0.66 | 2.32 |
| O34 Maternal care for known or suspected abnormality of pelvic organs | ID vs gPop | 0.66 | 0.43 | 1.03 | 0.91 | 0.58 | 1.41 | 0.84 | 0.54 | 1.30 | 0.80 | 0.51 | 1.25 |
|  | Mild ID vs gPop | 0.64 | 0.33 | 1.22 | 0.93 | 0.48 | 1.79 | 0.86 | 0.45 | 1.67 | 0.81 | 0.42 | 1.56 |
| O35 Maternal care for known or suspected fetal abnormality and damage | ID vs gPop | **1.91** | **1.13** | **3.24** | **2.09** | **1.23** | **3.54** | 1.52 | 0.89 | 2.60 | 1.34 | 0.78 | 2.28 |
|  | Mild ID vs gPop | **2.33** | **1.16** | **4.68** | **2.59** | **1.29** | **5.20** | 1.85 | 0.92 | 3.73 | 1.53 | 0.76 | 3.10 |
| O36 Maternal care for other known or suspected fetal problems | ID vs gPop | **1.41** | **1.15** | **1.72** | **1.27** | **1.04** | **1.55** | 1.21 | 0.99 | 1.48 | 1.14 | 0.93 | 1.39 |
|  | Mild ID vs gPop | 1.32 | 0.97 | 1.78 | 1.16 | 0.86 | 1.57 | 1.10 | 0.81 | 1.50 | 1.02 | 0.75 | 1.38 |
| O40 Polyhydramnios | ID vs gPop | 1.43 | 0.59 | 3.46 | 1.72 | 0.71 | 4.16 | 1.60 | 0.65 | 3.89 | 1.31 | 0.54 | 3.21 |
| O41 Other disorders of amniotic fluid and membranes | ID vs gPop | **1.61** | **1.07** | **2.42** | 1.43 | 0.95 | 2.16 | 1.32 | 0.87 | 2.00 | 1.28 | 0.84 | 1.94 |
|  | Mild ID vs gPop | 0.90 | 0.40 | 2.00 | 0.78 | 0.35 | 1.73 | 0.71 | 0.32 | 1.59 | 0.69 | 0.31 | 1.55 |
| O42 Premature rupture of membranes | ID vs gPop | 1.57 | 0.89 | 2.77 | 1.57 | 0.89 | 2.78 | 1.61 | 0.91 | 2.86 | 1.52 | 0.86 | 2.71 |
|  | Mild ID vs gPop | **2.51** | **1.30** | **4.84** | **2.52** | **1.31** | **4.86** | **2.58** | **1.33** | **5.01** | **2.42** | **1.24** | **4.69** |
| O46 Antepartum hemorrhage, not elsewhere classified | ID vs gPop | **1.64** | **1.08** | **2.50** | **1.67** | **1.10** | **2.54** | **1.54** | **1.00** | **2.35** | 1.46 | 0.95 | 2.23 |
|  | Mild ID vs gPop | 1.76 | 0.97 | 3.18 | 1.79 | 0.99 | 3.24 | 1.63 | 0.90 | 2.97 | 1.54 | 0.85 | 2.80 |
| O47 False labor | ID vs gPop | **1.77** | **1.47** | **2.13** | **1.56** | **1.29** | **1.88** | **1.37** | **1.13** | **1.65** | **1.27** | **1.05** | **1.53** |
|  | Mild ID vs gPop | **1.87** | **1.44** | **2.44** | **1.60** | **1.23** | **2.09** | **1.40** | **1.07** | **1.82** | 1.28 | 0.98 | 1.67 |
| O48 Prolonged pregnancy | ID vs gPop | **0.48** | **0.26** | **0.90** | **0.50** | **0.27** | **0.93** | 0.54 | 0.29 | 1.01 | 0.58 | 0.31 | 1.08 |
| **Complications of labor and delivery (O60-O75)** |  |  |  |  |  |  |  |  |  |  |  |  |  |
| O60 Preterm labor and delivery | ID vs gPop | 1.41 | 0.96 | 2.08 | 1.40 | 0.95 | 2.06 | 1.29 | 0.88 | 1.91 | 1.14 | 0.77 | 1.68 |
|  | Mild ID vs gPop | 1.39 | 0.79 | 2.45 | 1.38 | 0.78 | 2.44 | 1.26 | 0.71 | 2.23 | 1.07 | 0.61 | 1.90 |
| O61 Failed induction of labor^4^ | ID vs gPop | **1.30** | **1.07** | **1.58** | **1.32** | **1.08** | **1.61** | **1.28** | **1.05** | **1.56** | 1.19 | 0.98 | 1.46 |
|  | Mild ID vs gPop | 1.25 | 0.93 | 1.68 | 1.27 | 0.95 | 1.71 | 1.23 | 0.91 | 1.66 | 1.13 | 0.84 | 1.52 |
| O62 Abnormalities of forces of labor | ID vs gPop | 0.91 | 0.67 | 1.22 | 0.86 | 0.64 | 1.15 | 0.95 | 0.71 | 1.28 | 0.96 | 0.72 | 1.30 |
|  | Mild ID vs gPop | 0.88 | 0.57 | 1.36 | 0.82 | 0.53 | 1.27 | 0.92 | 0.59 | 1.43 | 0.94 | 0.61 | 1.46 |
| O64 Obstructed labor due to malposition and malpresentation of fetus | ID vs gPop | 1.63 | 0.85 | 3.15 | 1.75 | 0.91 | 3.38 | 1.75 | 0.90 | 3.40 | 1.73 | 0.89 | 3.37 |
| O68 Labor and delivery complicated by fetal stress [distress] | ID vs gPop | 1.14 | 0.81 | 1.60 | 1.13 | 0.80 | 1.59 | 1.23 | 0.87 | 1.74 | 1.23 | 0.87 | 1.74 |
|  | Mild ID vs gPop | 1.03 | 0.61 | 1.74 | 1.02 | 0.61 | 1.73 | 1.12 | 0.66 | 1.90 | 1.12 | 0.66 | 1.89 |
| O70 Perineal laceration during delivery^5^ | ID vs gPop | 0.84 | 0.67 | 1.05 | **0.78** | **0.62** | **0.97** | 0.94 | 0.75 | 1.17 | 0.96 | 0.77 | 1.20 |
|  | Mild ID vs gPop | 0.81 | 0.58 | 1.12 | 0.73 | 0.53 | 1.01 | 0.90 | 0.65 | 1.25 | 0.93 | 0.67 | 1.29 |
| O71 Other obstetric trauma | ID vs gPop | 1.00 | 0.52 | 1.92 | 0.82 | 0.43 | 1.58 | 0.96 | 0.49 | 1.85 | 0.97 | 0.50 | 1.88 |
|  | Mild ID vs gPop | 1.65 | 0.79 | 3.48 | 1.31 | 0.62 | 2.75 | 1.54 | 0.73 | 3.25 | 1.57 | 0.74 | 3.33 |
| O72 Postpartum hemorrhage | ID vs gPop | 0.72 | 0.44 | 1.18 | 0.70 | 0.43 | 1.14 | 0.76 | 0.46 | 1.24 | 0.72 | 0.44 | 1.18 |
|  | Mild ID vs gPop | 0.58 | 0.26 | 1.29 | 0.56 | 0.25 | 1.24 | 0.61 | 0.27 | 1.36 | 0.57 | 0.25 | 1.27 |
| O75 Other complications of labor and delivery, not elsewhere classified | ID vs gPop | 1.05 | 0.81 | 1.36 | 1.05 | 0.81 | 1.37 | 1.11 | 0.85 | 1.44 | 1.12 | 0.86 | 1.46 |
|  | Mild ID vs gPop | 1.18 | 0.82 | 1.69 | 1.19 | 0.83 | 1.70 | 1.25 | 0.87 | 1.80 | 1.28 | 0.89 | 1.83 |
| **Mode of delivery (O80-O84)** |  |  |  |  |  |  |  |  |  |  |  |  |  |
| O80 Singel spontaneous delivery | ID vs gPop | 0.98 | 0.87 | 1.10 | 0.95 | 0.84 | 1.07 | 0.94 | 0.84 | 1.06 | 0.96 | 0.85 | 1.08 |
|  | Mild ID vs gPop | 0.98 | 0.83 | 1.17 | 0.94 | 0.80 | 1.12 | 0.94 | 0.79 | 1.11 | 0.96 | 0.80 | 1.14 |
| O81 Single delivery by forceps and vacuum extractor | ID vs gPop | 1.02 | 0.70 | 1.47 | 0.96 | 0.66 | 1.40 | 1.17 | 0.80 | 1.70 | 1.19 | 0.82 | 1.73 |
|  | Mild ID vs gPop | 1.24 | 0.76 | 2.03 | 1.16 | 0.71 | 1.90 | 1.43 | 0.87 | 2.34 | 1.47 | 0.90 | 2.41 |
| O82 Single delivery by caesarean section | ID vs gPop | 1.04 | 0.82 | 1.33 | 1.23 | 0.96 | 1.56 | 1.21 | 0.95 | 1.54 | 1.12 | 0.87 | 1.43 |
|  | Mild ID vs gPop | 0.95 | 0.65 | 1.37 | 1.15 | 0.79 | 1.67 | 1.13 | 0.78 | 1.64 | 1.03 | 0.71 | 1.49 |
| O82.0 Delivery by elective caesarean section | ID vs gPop | 0.95 | 0.59 | 1.52 | 1.22 | 0.76 | 1.96 | 1.23 | 0.76 | 1.99 | 1.15 | 0.71 | 1.86 |
|  | Mild ID vs gPop | 1.19 | 0.64 | 2.21 | 1.61 | 0.87 | 3.01 | 1.65 | 0.88 | 3.08 | 1.50 | 0.80 | 2.80 |
| O82.1 Delivery by emergency caesarean section | ID vs gPop | 1.19 | 0.89 | 1.61 | 1.28 | 0.95 | 1.72 | 1.25 | 0.93 | 1.68 | 1.17 | 0.87 | 1.58 |
|  | Mild ID vs gPop | 0.87 | 0.52 | 1.44 | 0.95 | 0.57 | 1.57 | 0.92 | 0.55 | 1.53 | 0.85 | 0.51 | 1.42 |

*^1^ Cohabitation status and education, ^2^ Pre-pregnancy chronic conditions including pulmonary hypertension, chronic renal disease, preexisting bleeding disorder, preexisting cardiac disease, HIV/AIDS, preexisting anemia, gastrointestinal disease, acute or moderate/severe asthma, substance use disorder, connective tissue or autoimmune disease, chronic hypertension, preexisting diabetes mellitus, neuromuscular disease, major mental health disorder, and thyrotoxicosis, ^3^ Excluding women with diabetes mellitus type 1 or type 2, ^4^ Excluding births with elective caesarean section, ^5^ Only vaginal births*
